# A doubling of stony coral cover on shallow forereefs at Carrie Bow Cay, Belize from 2014 to 2019

**DOI:** 10.1038/s41598-021-96799-2

**Published:** 2021-09-28

**Authors:** Luis X. de Pablo, Jonathan S. Lefcheck, Leah Harper, Valerie J. Paul, Scott Jones, Ross Whippo, Janina Seemann, David I. Kline, J. Emmett Duffy

**Affiliations:** 1 Amherst College, Amherst, MA 01002 USA; 2 Tennenbaum Marine Observatories Network, MarineGEO, Smithsonian Environmental Research Center, Edgewater, MD 21037 USA; 3 Smithsonian Marine Station, Fort Pierce, FL 34949 USA; 4 Oregon Institute of Marine Biology, Charleston, OR 97420 USA; 5 Smithsonian Tropical Research Institute, Ancón, Panama; 6 Zukunft-Umwelt-Gesellschaft (ZUG) gGmbH, International Climate Initiative, Berlin, Germany

**Keywords:** Marine biology, Tropical ecology, Biodiversity, Biodiversity, Community ecology, Ecological modelling

## Abstract

To better understand the decline of one of earth’s most biodiverse habitats, coral reefs, many survey programs employ regular photographs of the benthos. An emerging challenge is the time required to annotate the large volume of digital imagery generated by these surveys. Here, we leverage existing machine-learning tools (CoralNet) and develop new fit-to-purpose programs to process and score benthic photoquadrats using five years of data from the Smithsonian MarineGEO Network’s biodiversity monitoring program at Carrie Bow Cay, Belize. Our analysis shows that scleractinian coral cover on forereef sites (at depths of 3–10 m) along our surveyed transects increased significantly from 6 to 13% during this period. More modest changes in macroalgae, turf algae, and sponge cover were also observed. Community-wide analysis confirmed a significant shift in benthic structure, and follow-up in situ surveys of coral demographics in 2019 revealed that the emerging coral communities are dominated by fast-recruiting and growing coral species belonging to the genera *Agaricia* and *Porites*. While the positive trajectory reported here is promising, Belizean reefs face persistent challenges related to overfishing and climate change. Open-source computational toolkits offer promise for increasing the efficiency of reef monitoring, and therefore our ability to assess the future of coral reefs in the face of rapid environmental change.

## Introduction

Coral reefs are extraordinarily diverse ecosystems that provide valuable economic and ecosystem services, including tourism, fisheries and protection of shorelines from storm damage^1–3^. However, reefs worldwide are increasingly threatened by anthropogenic stressors including pollution, climate change, and overfishing. Depletion of herbivorous fishes can allow benthic micro- and macroalgae to proliferate, leading to “phase shifts” from coral- to algal-dominated states^1,4–6^. High algal cover can interfere with the recruitment, survivorship, and growth of corals, making it more difficult for reefs to return to a coral-dominated state, especially under the continued exploitation of herbivore communities^7^. These shifts are most evident in regions with a long history of overfishing and other impacts, such as in the Caribbean where a combination of stressors has led to a decline of over 80% in average hard coral coverage between the 1970s and early 2000s^8^.

In an effort to understand the long-term trajectories of coral reef ecosystems, a number of large-scale reef monitoring programs have emerged, such as CARICOMP, Atlantic and Gulf Rapid Reef Assessment, Reef Life Survey, and the Global Coral Reef Monitoring Network^9–12^. These monitoring programs provide a standardized suite of protocols to track changes on reefs across locations and scales, and many use benthic photoquadrats to quantify the cover of corals and other benthic organisms. Photoquadrats are images of the seafloor that provide a permanent record of benthic cover and composition^10,11^. Photoquadrats have several advantages over in situ diver surveys in terms of reduced field time and reproducibility, however, they also have notable drawbacks: after the photos are taken, they must be manually edited and scored to produce useful information, leading to an analytical bottleneck. Images that are poorly cropped, angled, focused and/or color balanced can further limit the precision of and greatly increase the time required to analyze the photos. This can be a result of unfavorable environmental conditions, limitations of the equipment used, and experience-level of the photographer.

**Figure 1:**
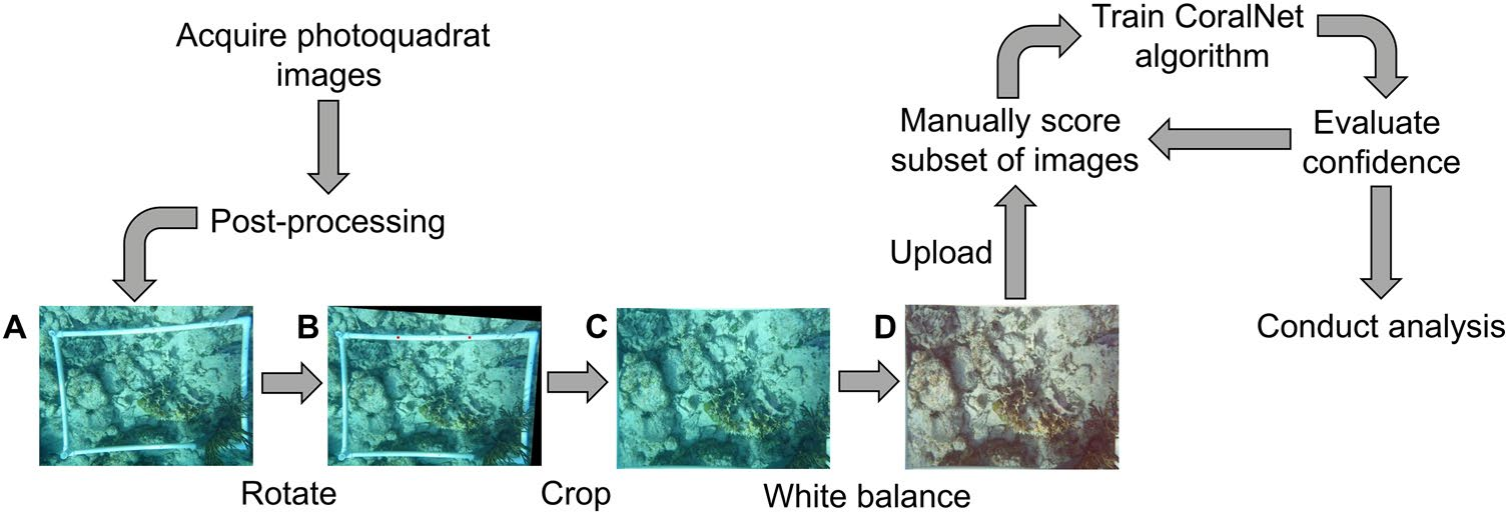
The workflow for the current study. Images of the benthos were taken and post-processed: (**A**) unedited photoquadrat; (**B**) the same photo after it was passed through a program designed to rotate the photo so that the PVC frame is square with the edges of the photo; (**C**) the photo cropped to the inside edge of the frame; and (**D**) the final, color-balanced version of the photoquadrat used for analysis. Post-processed images were uploaded to CoralNet, a subset of which were manually scored to train the algorithm. Images with low confidence were manually scored until all scores were within 50% confidence, which were then used to conduct the analysis.

As with many percent cover techniques, benthic photoquadrats are typically scored by identifying the organisms underneath a set number of points randomly distributed across the image^13^. This approach is faster and more objective than having to manually delineate the area of each organism, especially for a large number of images. The emergence of machine learning applications, such as CoralNet^14–16^, have further promoted the automated annotation of photoquadrat imagery^17^. Using these platforms, investigators manually train a machine-learning algorithm (e.g., Deep Learning Convolutional Neural Networks or Support Vector Machines) on a subset of images to recognize pre-defined benthic classes based on the texture and color of pixels under the point and of surrounding pixels (Fig. 1)^18^. The degree to which the investigator must train the model depends on the number, identity, and rarity of classifiers and the number of images, but previous applications of CoralNet have reduced human effort by 50–90% without appreciable loss of accuracy in genus or species identification^16^.

In this study, we leverage computer vision and machine learning methods to eliminate some of the major limitations of using photoquadrat surveys, including preparing and then annotating imagery. We use an example 5-year dataset of nearly 1000 photoquadrat images taken at Carrie Bow Cay, Belize as part of the Smithsonian Marine Global Earth Observatory (MarineGEO) long-term benthic monitoring program. We developed novel image correction software to standardize images prior to analysis. We then used machine learning, specifically Convolutional Neural Networks provided by CoralNet, to automatically classify benthic cover of major functional groups (Fig. 1). Carrie Bow Cay hosts a Smithsonian field station with a long history of coral reef research going back to the late 1960s^19–21^, and, as of 2020, Belize had the highest coral cover of any country in the Mesoamerican region^22^, making it an ideal region to study coral dynamics. Due to general improvements in both the management and ecology noted in the region, including increased protections for herbivorous fish and implementation of marine protected areas, we wished to track trends in cover of hard corals and macroalgae, especially in relation to the Caribbean-wide historical phase shift from corals to macroalgal dominance^23^.

## Methods

### Site description

**Figure 2:**
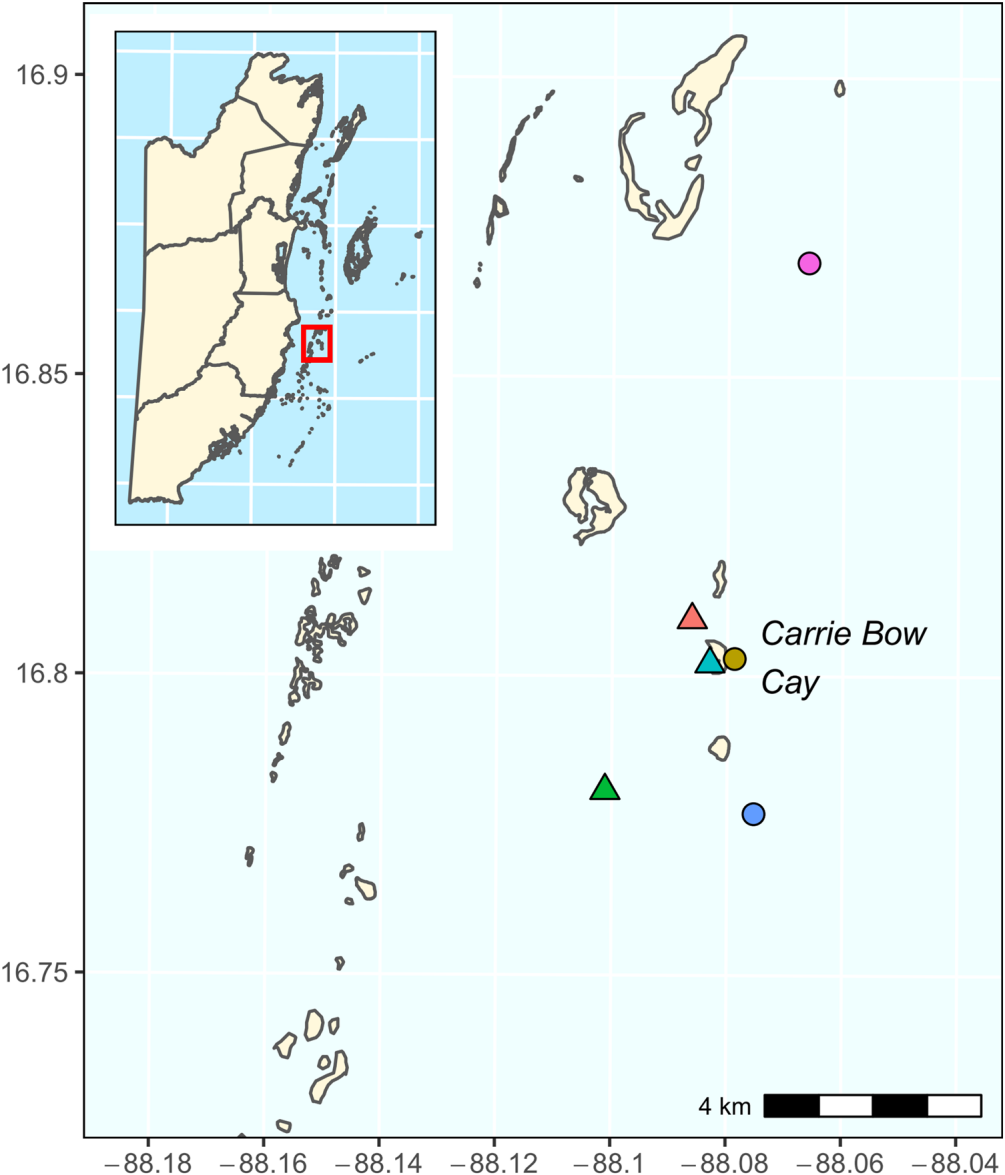
A map of the study area including the six locations (colored points) surveyed at Carrie Bow Cay, Belize. The country of Belize is shown as an inset, with the red box denoting the area of the larger image. Colors and symbols correspond to reef sites/types in the legend of Fig. 3. To generate the map, we used the *sp*^24^ and *sf*^25^ packages in R version 4.0.5^26^.

Carrie Bow Cay lies within the Southwater Caye Marine Reserve approximately 20 km off the coast of Belize on the Mesoamerican Reef (MAR). The MAR stretches from the edge of the Yucatan Peninsula in the north, to the eastern edge of Honduras to the south. It is the largest barrier reef in the Western Hemisphere. Data were collected from 6 shallow-water (3–10 m) localities at Carrie Bow Cay separated by 0.5–10.5 km, including: two patch reefs (CBC Lagoon Reef 16.8086° N, 88.0862° W; and Curlew Patch Reef, 16.7800° N, 88.1012° W), three forereefs (CBC Reef Central, 16.8021° N, 88.0788° W; South Reef Central, 16.7762° N, 88.0754° W; Tobacco Reef, 16.8683° N, 88.0662° W); and fringing reef we classified as more like the patch reefs (CBC House Reef, 16.8013° N, 88.0831° W) (Fig. 2). Data were collected from one 50-m transect at each of the 6 reef sites.

**Figure 3:**
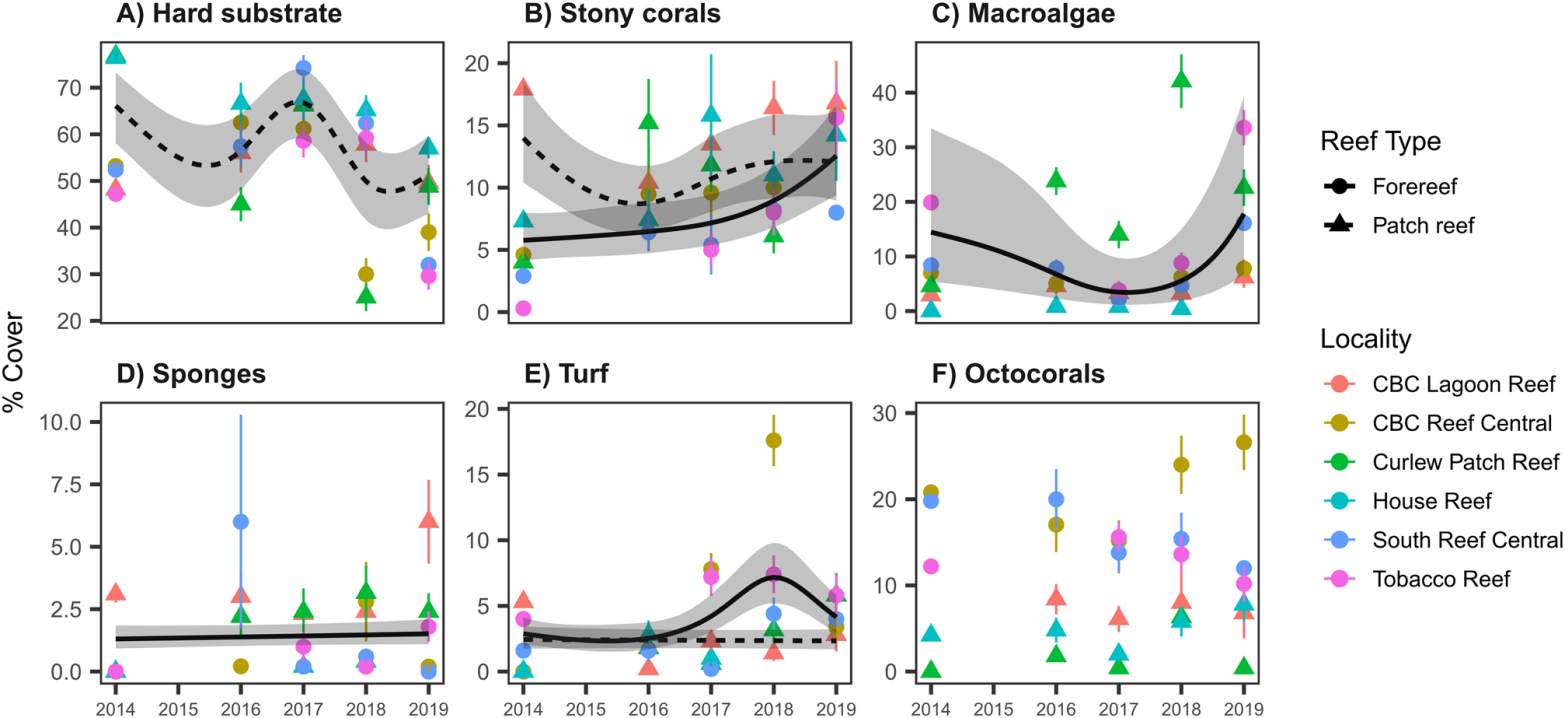
Mean percent cover for each of the five main benthic functional groups and consolidated (hard) substrate. Points are means ± 1 standard error for each of the 6 localities. Lines are fitted predictions from a generalized additive model ± 95% confidence intervals for only those reef types with significant (*P* < 0.05) changes in cover through time, with solid lines denoting significant trends on patch reefs and dashed lines on forereefs.

### Collection of photoquadrats

We followed the protocol for acquiring photoquadrat imagery from the Reef Life Survey network^9^. Briefly, at each site at a depth of 3–5 m, a single 50-m transect was laid across the reef, and an image was taken by a trained SCUBA diver every 2.5-m, approximately 0.5-m above the benthos, thereby covering an average area of 0.74 ± 0.50 m^2^ per image. A minimum of 20 photoquadrats were collected from each site in 2014, 2016, 2017, 2018, and 2019.

Two localities, CBC Reef Central and South Reef Central, were surveyed using fixed transects laid out in the same place each year. The other four localities used random transects placed in a different location within the site each year. While fixed transects offer a higher precision than random transects, enabling the detection of smaller changes, random transects provide data are more reflective of general reef-level changes.

### Preparation of photoquadrats

Photoquadrat images were first examined manually to remove any duplicate images and to ensure that there were 20 photos per year from each location. In 2014, upwards of 80 images were taken at each site, so we used a randomized resampling procedure to generate median percent cover values for *n* = 20 replicate images across 1000 iterations for each location during that year (see Supplementary Code).

Photoquadrats from 2014 contained a PVC frame intended to standardize the area contained in each photo that was dropped for later surveys. For these images, we used another purpose-built computer vision program in Java to crop the image to the inside edge of the frame (https://github.com/lxdepablo/quadratCropper). Photos were subsequently passed through a purpose-built color-balancing program using Java to aid the human annotator (https://github.com/lxdepablo/colorBalancer). This program worked by finding the average green and blue values of all the pixels in an image, and then raising the red value in proportion to the average green and blue values. The program also decreased the brightness of every pixel by a fraction of the overall brightness to correct overexposed images to aid in manual annotations.

### Analyzing photoquadrats

Photoquadrats were analyzed using CoralNet (https://coralnet.ucsd.edu/)^14,15^. The automated annotator was trained on 228 images with 10 points per image for a total of 2280 manually scored points. We then generated 25 random points per image on the 20 images at each of the six localities in each year of the survey, and a machine annotator automatically scored the functional group under each point. Points where the machine annotator had over 50% confidence were scored automatically, and the rest of the points with < 50% confidence were manually scored by one human annotator (Fig. 2). The final confidence level was 92% through a combination of manual and automated classification. All images throughout the study were scored by the same annotator to avoid inter-operator differences.

Functional groups within CoralNet were classified using the standard CATAMI classification scheme^27^. Labels included macroalgae, long turfing algae, scleractinian (stony) corals, octocorals, sponges, sand, and rubble^28^. We added another category which we called “consolidated substrate” capturing the diverse consortium of filamentous algae, crustose coralline algae, and other microorganisms also referred to as the “epilithic algal matrix” on which sessile organisms can potentially settle^29^. These broad labels were selected to achieve maximum accuracy from the machine learning models with minimum training and bias from the human annotator^16^. Several labels from outside the CATAMI scheme were added to enhance quality. These included labels for objects obscured by shadow, the transect tape, and unidentifiable objects, based on a protocol recommended by the Pacific Islands Fisheries Science Center^30^.

### In situ coral surveys

The species-level demographics of the scleractinian coral community were also assessed in situ at each site in 2019 using transect survey methods adapted from the IUCN Resilience Assessment of Coral Reefs Handbook^31^. Along each 50 m transect, a 30-x-1 m belt was surveyed. All scleractinian corals > 1 cm in diameter with live tissue falling within the belt were identified to species level and binned by size class. More details on the survey design and classifications are available in Obura and Grimsditch^31^.

### Statistical analysis

In the years before the implementation of a standardized photoquadrat, the images collected captured varying areas of the seafloor (median = 0.72 m^2^). Therefore, we conducted an analysis to determine if benthic cover of each of our categories was influenced by the area of bottom captured in the images. First, we computed the area of three randomly selected images from each locality in 2016, 2017, and 2018 that had a legible scale bar present in the image (*n* = 42 images). We then computed the area of the image using the ImageJ software (v1.52 from https://imagej.nih.gov/)^32^. Finally, we used simple linear regression of percent cover obtained from the machine scoring for each major benthic group with > 1% cover against the log_10_-transformed area of the corresponding image. The results of this analysis showed cover was not significantly related to image area for any of the major benthic groups (0.20 < *P* < 0.99) (Supplementary Fig. S1). Therefore, in the absence of any detectable systematic bias due to image area, we chose to proceed with the raw (unscaled) percent cover values in our analysis.

To analyze temporal trends in percent cover of the major benthic categories, we employed generalized additive models (GAMs)^33^. GAMs are ideal for identifying and modeling potentially non-linear changes through the use of non-parametric smoothing functions. In the case of our data, we analyzed the cover of six main benthic categories with sufficient representation (> 1% average cover across all images): scleractinian corals, octocorals, macroalgae, turf algae, sponges, and consolidated (hard) substrate. For each response, we fit a smoothed term for year, for each reef type by year (i.e., a separate smoothed function for forereef and patch reefs through time), a parametric effect of reef type (patch vs. forereef), and a random effect of locality to account for reef-to-reef variability in cover.

Recognizing that proportional cover is bound between 0 and 1, we fit each GAM to a beta distribution. Because this distribution cannot accommodate true zero observations (i.e., those where the benthic category is actually absent rather than just unobserved), we transformed the response *p* using Eq. 1 to shift the bounds to include [0, 1] following^31^:

1 $${p}^{*}=\frac{p\left(n-1\right)+0.5}{n},$$

where *n* is the total number of observations. Model assumptions (i.e., normality of errors) were assessed visually.

To visualize changes in whole-community structure through time, we employed non-metric multidimensional scaling (NMDS) on the square-root transformed average percent cover of each functional group in each locality in each year. To assess the significance of changes in community structure, we used redundancy analysis (RDA) of the community response matrix used in the NMDS analysis against the independent variables of location and year, and then applied a permutation-based ANOVA to assess significance (n = 999). To conduct the analyses, we used the *mgcv*^33^, *mgcViz*^34^, and *vegan*^35^ packages in R version 4.0.5^26^. All data and code for statistical analyses are provided in the Supplementary Information.

## Results

Consolidated hard substrate comprised most of the benthic cover in all years and significantly declined from 66 ± 0.2% in 2014 to 51 ± 0.2% in 2019 based on output from the generalized additive model (*P* < 0.001; Fig. 3A; Table 1). Over the same period, scleractinian coral cover more than doubled from an average of 5.8 ± 0.2% to 13 ± 0.2%, but only at the forereef localities (*P* < 0.001; Fig. 3B; Table 1). In contrast, the temporal trend in scleractinian coral cover on patch reefs was more complicated, despite coral cover being overall higher than on forereefs (*P* = 0.032; Table 1). At these localities, coral cover initially declined, dipping to 8.8 ± 0.2% in 2016, but returned to 12 ± 0.2% by the 2019, which was similar to the coral cover observed in 2014 (*P* = 0.007; Table 1; Fig. 3B).

**Table 1:** Results from generalized additive models predicting the six main benthic categories. Critical value is *Z-*score (parametric) and χ^2^ (non-parametric tests).

Response	Predictor	Deviance explained	Type	Critical value	P-value
Hard substrate	(Intercept)	33.3%	Parametric	2.2544	0.0242*
Hard substrate	Reef type [forereef]		Parametric	− 1.2741	0.2026
Hard substrate	s(Year)		Non-parametric	96.9696	< 0.0001***
Hard substrate	s(Year):Reef type [patch reef]		Non-parametric	44.1994	< 0.0001***
Hard substrate	s(Year):Reef type [forereef]		Non-parametric	0.0001	0.9911
Hard substrate	s(Locality)		Non-parametric	52.5740	< 0.0001***
Stony corals	(Intercept)	15.9%	Parametric	− 15.1774	< 0.0001***
Stony corals	Reef type [forereef]		Parametric	− 2.1443	0.0320*
Stony corals	s(Year)		Non-parametric	4.2064	0.0406*
Stony corals	s(Year):Reef type [patch reef]		Non-parametric	13.5926	0.0066**
Stony corals	s(Year):Reef type [forereef]		Non-parametric	17.7829	0.0008**
Stony corals	s(Locality)		Non-parametric	21.2498	< 0.0001***
Macroalgae	(Intercept)	62.6%	Parametric	− 5.1826	< 0.0001***
Macroalgae	Reef type [forereef]		Parametric	0.5082	0.6113
Macroalgae	s(Year)		Non-parametric	18.6130	0.0011**
Macroalgae	s(Year):Reef type [patch reef]		Non-parametric	7.9335	0.0736
Macroalgae	s(Year):Reef type [forereef]		Non-parametric	6.7361	0.0380*
Macroalgae	s(Locality)		Non-parametric	496.8929	< 0.0001***
Sponges	(Intercept)	21.0%	Parametric	− 27.0877	< 0.0001***
Sponges	Reef type [forereef]		Parametric	− 1.2965	0.1948
Sponges	s(Year)		Non-parametric	0.6098	0.4350
Sponges	s(Year):Reef type [patch reef]		Non-parametric	< 0.0001	1.0000
Sponges	s(Year):Reef type [forereef]		Non-parametric	14.6301	0.0001***
Sponges	s(Locality)		Non-parametric	24.6826	< 0.0001***
Turf	(Intercept)	9.3%	Parametric	− 25.1143	< 0.0001***
Turf	Reef type [forereef]		Parametric	2.5378	0.0112*
Turf	s(Year)		Non-parametric	17.1118	< 0.0001***
Turf	s(Year):Reef type [patch reef]		Non-parametric	15.6189	0.0001**
Turf	s(Year):Reef type [forereef]		Non-parametric	30.7867	< 0.0001***
Turf	s(Locality)		Non-parametric	24.1210	< 0.0001***
Octocorals	(Intercept)	48.2%	Parametric	− 22.0211	< 0.0001***
Octocorals	Reef type [forereef]		Parametric	7.0993	< 0.0001***
Octocorals	s(Year)		Non-parametric	1.9680	0.1609
Octocorals	s(Year):Reef type [patch reef]		Non-parametric	0.0001	0.9940
Octocorals	s(Year):Reef type [forereef]		Non-parametric	13.5345	0.0020**
Octocorals	s(Locality)		Non-parametric	21.5043	< 0.0001***

Macroalgal cover exhibited a similar non-linear trend through time at forereef localities (*P* = 0.038; Table 1), where cover was reduced until 2017 and then rose to its initial average cover of 18 ± 0.6% in 2019 (Fig. 3C). Two other benthic groups also significantly increased at forereef sites: sponges almost imperceptibly given the range of variation, and turfs to a slightly greater extent (Fig. 3D,F; Table 1), although these increases were much smaller and only reflected < 5% of the benthos in any given year. Finally, octocorals maintained consistently low benthic cover on patch reefs (0–8.4%) and consistently high cover on forereefs (10–26%) that did not significantly change over the survey period (Table 1; Fig. 3F).

These general trends in benthic cover obscure variability among the localities, with some maintaining high coral cover (~ 15% at CBC Lagoon Reef, Fig. 3B) and others reaching high macroalgal cover (~ 40% at Curlew Patch Reef in 2018, Fig. 3C). Nevertheless, four out of the six individual sites surveyed showed positive trajectories in living coral cover, including the two locations with fixed transects (Supplementary Fig. S2). This result suggests that our main findings are not entirely due to slight deviations in transect position between surveys but instead reflect real increases in coral cover, especially on forereefs. Indeed, if we re-run our GAM restricting it to only the two fixed transects, there remains a significant increase in scleractinian coral cover through time (*P* = 0.010). Similarly, the curvilinear decline and subsequent increase in macroalgae was also observed and statistically significant at these two fixed sites (*P* < 0.001).

**Figure 4:**
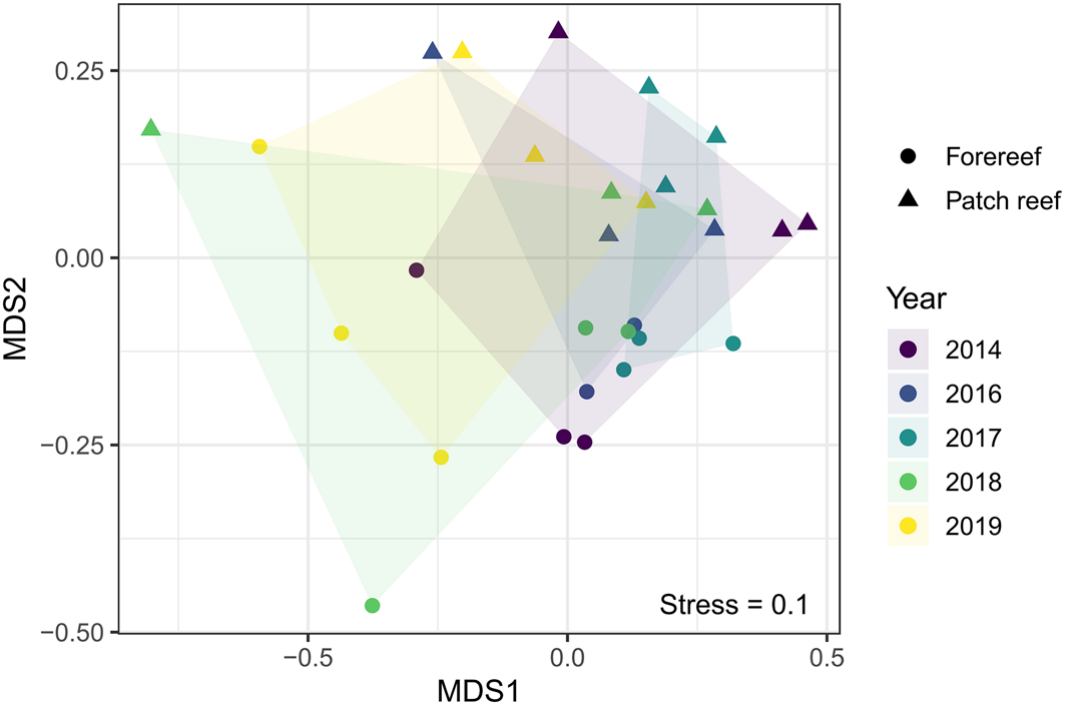
Spatial and temporal variation in benthic community composition visualized using non-metric multidimensional scaling. Points correspond to each locality in each year, and shaded areas are convex hulls for each year of the survey. Stress (a measure of agreement between the original multivariate and reduced dimensionality) is give in the lower right corner (0.1 < stress < 0.2 = good to excellent fit).

These general temporal changes were corroborated in our NMDS analysis, which revealed a shift in overall community structure from 2014 to 2019 driven by communities at both patch and forereef sites based on data from the CoralNet automated classifier (Fig. 4). ANOVA of the RDA confirmed significant effects of reef type, locality, and year on benthic community composition (*P* < 0.001 in all cases). Our in situ demographic survey revealed that most of the scleractinian corals observed at the end of the study period belonged to weedy or opportunistic species in the genera *Agaricia* and *Porites*, fast-growing corals such as *Acropora cervicornis* that contribute to vertical reef structure (found only at Tobacco forereef), and stress-tolerant species in the genus *Siderastrea* (Supplementary Fig. S3).

## Discussion

Our results show that scleractinian corals belonging to a variety of genera doubled in cover between 2014 and 2019 across three shallow-water forereefs around Carrie Bow Cay, Belize and even exceeded macroalgal cover on two patch reefs (CBC Lagoon Reef and CBC Reef Central; Fig. 3). Together, these results suggest a recent positive trajectory for coral cover at Carrie Bow Cay, Belize, mirroring the general pattern of recovery recently noted across the region^22^ and on other Caribbean reefs^36–38^. Further, we provide a pipeline for image processing (Fig. 1) and new open-source programs to aid in preparing benthic imagery for human annotation and subsequent automated image analysis.

There are several possible explanations for the increase in coral cover we observed around Carrie Bow Cay. First, macroalgal cover was overall quite low at Carrie Bow Cay (mean of 8.4% across all sites and years) compared to other locations along the MesoAmerican Reef, such as Mexico (approximately 25% cover)^36^ and Honduras (20–25% cover)^39^. As a result, reefs at Carrie Bow might have been better positioned than these other locations to observe coral recovery, as higher macroalgal cover has been shown to both deter coral recruits and hinder coral juvenile survivorship^40,41^.

Second, and relatedly, herbivorous fish density is relatively high in Belize compared to other reefs in the Caribbean, and fish biomass has been shown to be increasing over the past decade across large portions of the MAR, in part due to fishing restrictions such as Belize’s ban on harvesting herbivorous fishes^23^ and the implementation of marine protected areas^39^. High herbivore biomass has been linked to greater consumption of macroalgae throughout the Caribbean^39,42,43^ and most recently in Southwater Caye Marine Reserve^44^, and may explain the overall lower abundance of macroalgae at our sites, keeping the substrate open for colonization by corals over the period of our survey^40^.

Third, the coral community at Carrie Bow Cay is currently dominated by so-called “weedy” or opportunistic coral species, such as those belonging to the genera *Agaricia* and *Porites*, based on our surveys conducted in 2019. These are common fast-recruiting individuals that make up an increasing proportion of coral cover throughout Belize and the Caribbean^45,46^. Because most species within these genera reach small adult sizes, one implication is that their rise and the concurrent loss of framework-building corals such as *Orbicella* spp. in Belize has led to reef “flattening”^47^, which may have implications for further recovery of fish communities requiring higher structural complexity^48^. However, the presence of *A. cervicornis*—a fast-growing reef-builder—at one of the forereef sites in 2019 suggests higher potential for recovery of three-dimensional complexity in these localities (Supplementary Fig. S3).

A major question is why neither corals nor macroalgae have exceeded > 20% cover on these reefs? One possibility is that the region is still recovering from several documented mass coral mortality events: first of *A. cervicornis* due to white-band disease in the 1980s^49^, and then of *Agaricia tenuifolia* due to elevated temperatures and hurricanes in 1998^50^. The loss of adult corals has obvious implications for coral recruitment, leading to slow recovery that has seemingly stretched over several decades^51^. Additionally, Carrie Bow Cay is relatively remote and not subject to human influences that might promote algal blooms, as has been seen in other parts of the MAR with the rise of tourism and development and which exhibit much higher macroalgal cover^52^.

The analysis pipeline created for this project—including the use of CoralNet automated classifiers, purpose-built computer programs, data collection and management practices, and overall workflow—can be applied to continued surveys at Carrie Bow Cay and other reefs. This project serves as a proof of concept for further larger-scale reef monitoring programs in the Caribbean and beyond. Moving forward, this pipeline could be fully automated using CoralNet’s public API, released in early 2020^53^. Images could be sorted, prepared, and analyzed with limited or no human intervention, thereby enabling analysis of even larger data sets and development of larger, more ambitious monitoring projects that will improve the conservation and management of coral reefs. While we resolved to the functional group level (e.g., stony corals, macroalgae, etc.), future refinements to the algorithm and improvements in computing power will likely extend classifications to the species-level.

One potential source of error in our survey is the mixing of fixed vs. random transects: two sites used fixed transects (CBC Reef Central and South Reef Central, both forereef sites) while the remaining four varied the position of the transects slightly year-to-year to accommodate local conditions on the day of the survey. While an ideal survey design would implement totally fixed transects to increase precision and therefore increase the chances of capturing small scale changes, we believe our results are generally robust for a few reasons. First, the two fixed transects are on forereef sites where stony coral recovery is notably higher from 2014 to 2019: + 176% for South Reef Central and + 243% for CBC Reef Central. Second, we witnessed an incredible increase in stony coral cover at Tobacco Reef, also a forereef site: + 5100% since 2014, a consequence of transitioning from almost no coral cover (< 0.01%) in 2014 to a substantial cover of primarily *A. cervicornis* by 2019 (15.6%). This observation—while potentially due to shifting the transect on the scale of a few meters, although unlikely given the magnitude of the percent change—also agrees with anecdotal observations from researchers who have been working in the region for many years to decades, and further aligns with regional recovery recently reported in Mumby et al.^44^.

Despite our findings of a doubling in primarily forereef coral cover over 5 years, Caribbean corals continue to face threats that imperil their recovery and long-term prospects. Warming, ocean acidification, hurricanes, and overfishing continue to be prominent threats to reef health in the region^38^. Of particular and growing importance is the stony coral tissue loss disease, which originated in Florida^54^ and continues to expand south through Mexico^55^, Puerto Rico^56^, and Turks and Caicos^57^. Stony coral tissue loss disease was confirmed in northern Belize by the country’s national Fisheries Department in 2019 and has now reached central Belize in 2021 (*pers. comm.*).When the disease reaches Carrie Bow Cay, it will surely alter the trends reported here, as this disease has been linked to massive die-offs in Florida and Mexico^55,58^. The imaging analysis pipeline described here will streamline continued coral reef monitoring that is crucial for both charting the future trajectory of corals at Carrie Bow Cay and recognizing the first instances of novel impacts such as stony coral tissue loss disease.

## Supplementary Information

Supplementary Information 1.

Supplementary Information 2.

Supplementary Information 3.

Supplementary Information 4.

Supplementary Information 5.

Supplementary Information 6.

Supplementary Information 7.

Supplementary Figures.

## Data Availability

The datasets and code used in this study are available as Supplementary Information.
