# Supplementary material for: A doubling of stony coral cover on shallow forereefs at Carrie Bow Cay, Belize from 2014 to 2019
Source: Sci Rep. 2021 Sep 28;11:19185. doi: 10.1038/s41598-021-96799-2 (PMC8478911; doi:10.1038/s41598-021-96799-2)
Supplement: Supplementary file 8 — Supplementary Figures. [file 41598_2021_96799_MOESM8_ESM.docx]

**A doubling of stony coral cover on shallow forereefs at Carrie Bow Cay, Belize from 2014-2019**

Luis X. de Pablo*^1^, Jonathan S. Lefcheck^†2^, Leah Harper^2^, Valerie J. Paul^3^, Scott Jones^3^, Ross Whippo^4^, Janina Seemann^5,6^, David I. Kline^5^, J. Emmett Duffy^2^

^1^Amherst College, Amherst, Massachusetts 01002

^2^Tennenbaum Marine Observatories Network, MarineGEO, Smithsonian Environmental Research Center, Edgewater, Maryland 21037

^3^Smithsonian Marine Station, Fort Pierce, Florida 34949

^4^Oregon Institute of Marine Biology, Charleston, Oregon 97420

^5^Smithsonian Tropical Research Institute, Ancón, Panama

^6^Zukunft-Umwelt-Gesellschaft (ZUG) gGmbH, International Climate Initiative, Germany

*Contact Email: [luisxdepablo@gmail.com](mailto:luisxdepablo@gmail.com)

^†^Contact Email: [LefcheckJ@si.edu](mailto:LefcheckJ@si.edu)

**Supplementary Material**


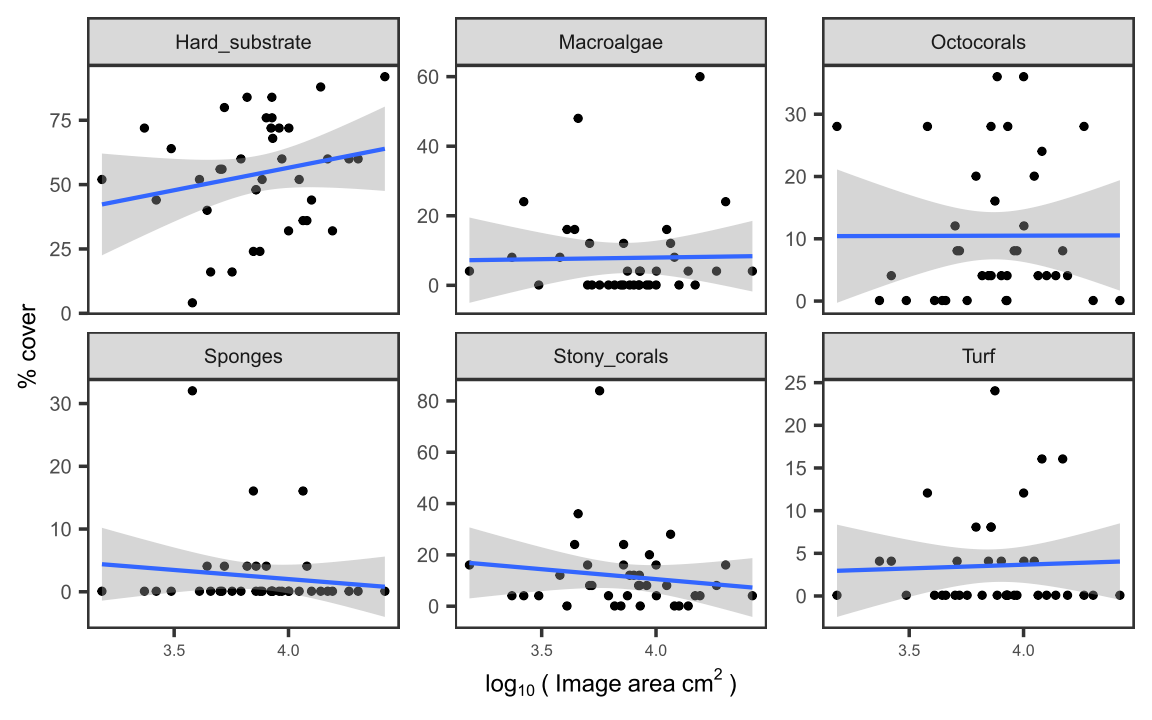


**Figure S1.** Tests of relationships between photoquadrat image area and cover of major benthic categories (>1% across all localities and years). Lines are predicted fits from simple linear regression with shaded area corresponding to + 95% confidence intervals.


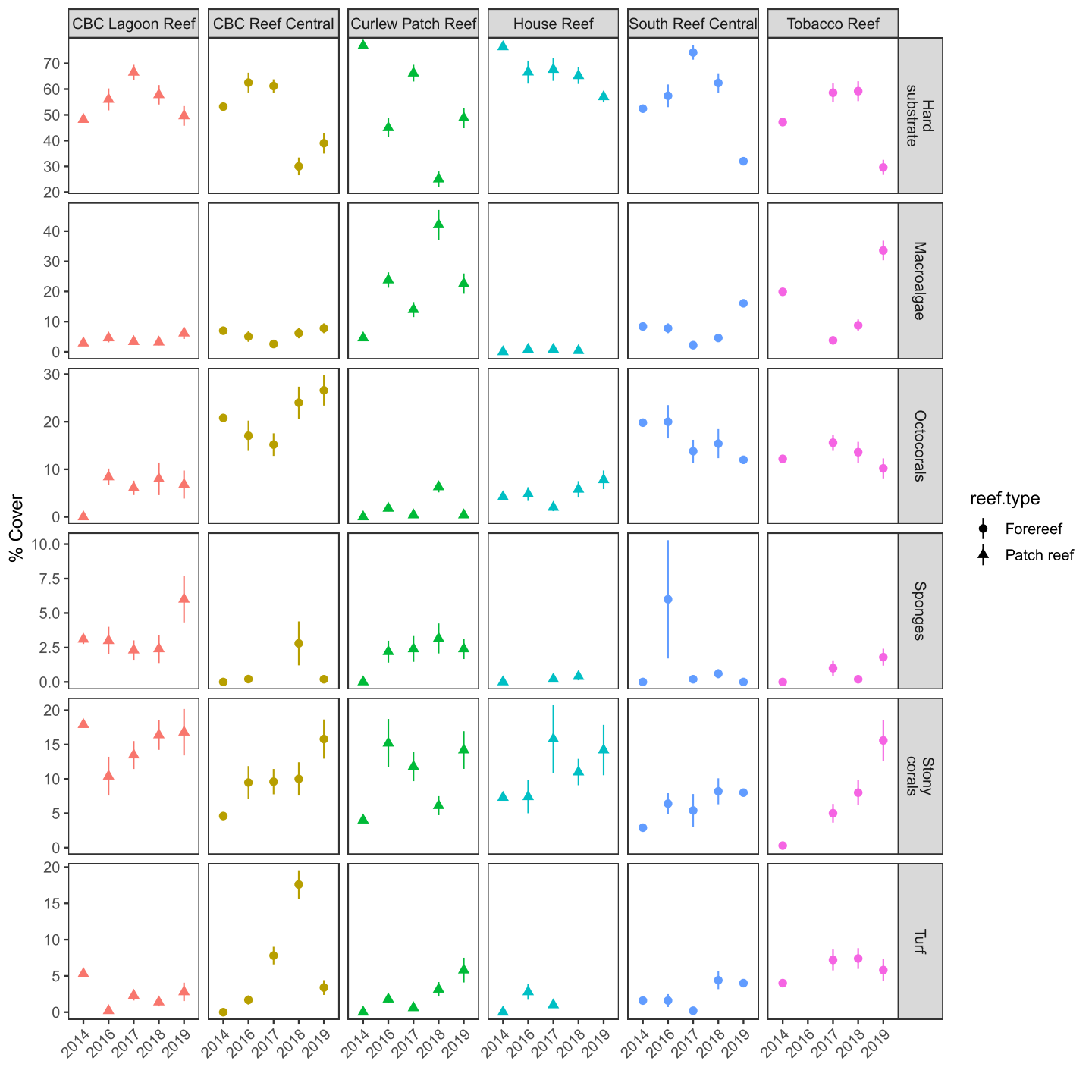


**Figure S2.** Site-specific trends in benthic cover for each of the six localities and the major benthic groups. Points are means + 1 standard error.


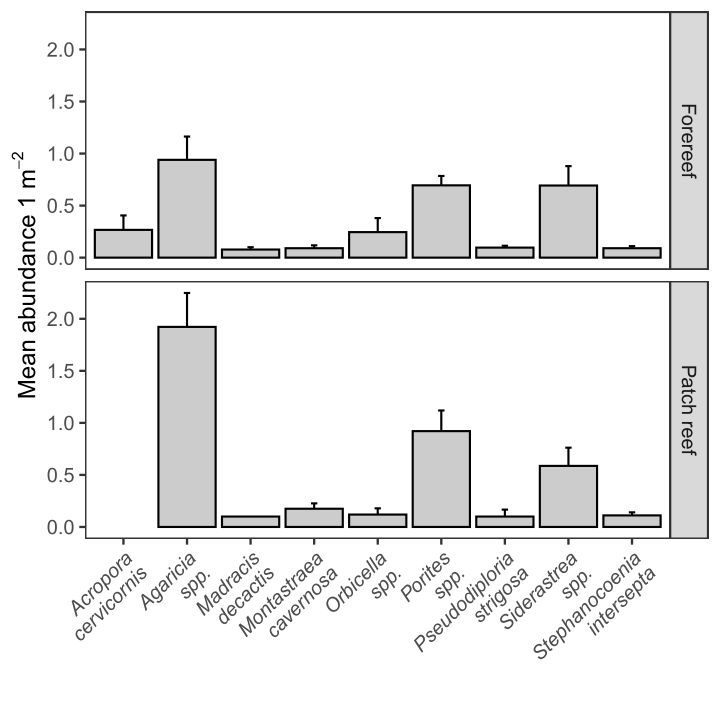


**Figure S3.** Average abundance + 1 standard error of stony coral genera from demographic surveys conducted in 2019.
